# Dynamic evolution of retroviral envelope-derived sequences in primates

**DOI:** 10.1093/ve/veag044

**Published:** 2026-07-16

**Authors:** Koichi Kitao, Kirill Kryukov, Lihua Jin, Yuta Shintaku, Takashi Hayakawa, So Nakagawa

**Affiliations:** Laboratory of Genome and Epigenome Dynamics, Department of Animal Sciences, Graduate School of Bioagricultural Sciences, Nagoya University, Furo-cho, Chikusa-ku, Nagoya 464-8601, Japan; Laboratory for Retrotransposon Dynamics, RIKEN Center for Integrative Medical Sciences, 1-7-22 Suehiro-cho, Tsurumi-ku, Yokohama, Kanagawa 230-0045, Japan; Center for Genome Informatics, Joint Support-Center for Data Science Research, Research Organization of Information and Systems, 1111 Yata, Mishima, Shizuoka 411-8540, Japan; Bioinformation and DDBJ Center, National Institute of Genetics, 1111 Yata, Mishima, Shizuoka 411-8540, Japan; Department of Molecular Life Science, Tokai University School of Medicine, 143 Shimokasuya, Isehara, Kanagawa 259-1193, Japan; Division of Omics Sciences, Institute of Medical Sciences, Tokai University, 143 Shimokasuya, Isehara, Kanagawa 259-1193, Japan; Japan Monkey Centre, 26 Kanrin, Inuyama, Aichi 484-0081, Japan; Wildlife Research Center, Kyoto University, 2-24 Tanaka-Sekiden-cho, Sakyo, Kyoto 606-8203, Japan; Faculty of Environmental Earth Science, Hokkaido University, North 10, West 5, Kita-ku, Sapporo, Hokkaido 060-0810, Japan; Department of Molecular Life Science, Tokai University School of Medicine, 143 Shimokasuya, Isehara, Kanagawa 259-1193, Japan; Division of Omics Sciences, Institute of Medical Sciences, Tokai University, 143 Shimokasuya, Isehara, Kanagawa 259-1193, Japan; Division of Interdisciplinary Merging of Health Research, Micro/Nano Technology Center, Tokai University, 4-1-1 Kitakaname, Hiratsuka, Kanagawa 259-1292, Japan

**Keywords:** endogenous retrovirus, *de novo* genes, co-option, primate evolution, comparative genomics

## Abstract

It is known that several endogenous retroviruses, remnants of ancient retroviral integrations, retain envelope (*env*) genes encoding fusogenic proteins in primates. Whilst most *env* genes are degraded, a few have been co-opted by hosts, yet the entire evolutionary dynamics of *env* sequences remain poorly understood. To explore this, we screened and compared *env* open reading frames (ORFs) from 247 primate genomes. In total, 8683 nearly intact *env*-ORFs encoding 400 or more amino acids were identified, and their copy numbers ranged from 3 to 429 across primate species. Sequence similarity clustering revealed a clear evolutionary signature that distinguishes the small set of long-term co-opted *env*-derived genes, which are retained at low copy numbers across lineages, from the broader pool of rapidly turning-over *env*-ORFs. Applying this framework, we identified a previously unrecognized, single-copy *env* ortholog conserved across Tarsiiformes, named *env-Tar1*, and experimentally demonstrated its cell fusion activity *in vitro*, providing the first functional evidence of an *env*-derived gene co-opted in a tarsier lineage. Further, we found that certain co-opted *env*-derived genes may have lost their functions due to nonsense or indel mutations within specific primate lineages. Considering that many *env* genes tend to be maintained at low copy numbers and are reported to be under purifying selection, such dynamic evolutionary turnover of *env*-derived genes may be driven by host-virus arms races, as viruses and endogenous retroviruses often share cell-surface receptors.

## Introduction

It is known that ~8% of the human genome consists of long terminal repeat (LTR) retrotransposons, primarily derived from endogenous retroviruses (ERVs) (Lander *et al*. 2001, Hoyt *et al*. 2022). ERVs are remnants of ancient retroviral infections that became integrated into the host germline genome and are inherited across generations (Johnson 2019). The typical integrated provirus consists of genes encoding capsid structural proteins (*gag*), retroviral enzymes (*pol*), and envelope glycoproteins (*env*) flanked by LTRs. Amongst them, 0.04% of ERVs retain open reading frames (ORFs) exceeding 80 amino acids in length (Ueda *et al*. 2020). Although this fraction appears small, the human genome contains 1731 *env*-derived ORFs encoding proteins longer than 80 amino acids (Nakagawa and Takahashi 2016). This number exceeds that of olfactory receptor genes, including pseudogenes, which constitute the largest multigene family in the human genome (Niimura and Nei 2003). Env proteins of retroviruses recognize cell receptors and fuse the viral and cellular membranes during infection. Whilst most *env* genes are truncated, some near-intact ORFs have been co-opted for new functions by leveraging their inherent molecular activity (Grandi and Tramontano 2018).

**Figure 1:**
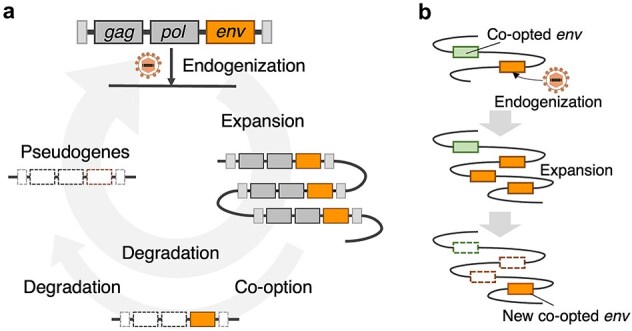
Dynamic evolution of env genes in host genomes. (a) The cycle of copy number change of retroviral *env* genes. Once ERV is integrated into the host genome by infection to germ cells, its copy number will increase by retrotransposition. However, mutation causes loss of retrotransposition ability, and the copy number increase is halted. Most *env* genes and other retroviral genes evolve neutrally and lose coding capacity through the accumulation of mutations, becoming pseudogenes. A small number of *env* genes are co-opted and conserved. However, co-opted *env* genes are sometimes lost. (b) Scheme of the baton pass hypothesis. For simplicity, only *env* genes are shown. When a new *env* gene is introduced and expanded in the genome, the selection pressure on the existing co-opted *env* gene is relaxed. Then, one of the new *env* genes takes over the function and is evolutionarily conserved.

Several Env proteins have acquired roles in placental development in primates. Syncytin-1 and Syncytin-2, although derived from different ERV *env* genes (*ERVW-1* and *ERVFRD-1,* respectively), promote syncytiotrophoblast cell fusion in Hominoidea (apes) species (Mi *et al*. 2000, Blaise *et al*. 2003, Gholami Barzoki *et al*. 2023, Priščáková *et al*. 2023). *ERVW-1* originated in the common ancestor of Catarrhini but lost function in many Cercopithecoidea (Old World monkey) species due to nonsense mutations (Bonnaud *et al*. 2005). *ERVFRD-1* arose in the Simiiformes (simian) ancestor (Blaise *et al*. 2003), but its protein appears non-fusogenic in some Platyrrhini (New World monkey) species (Shoji *et al*. 2023). *ERVV-2* encodes Mac-syncytin-3, which shows placental fusogenicity in several Cercopithecoidea and Platyrrhini species, but not in Hominoidea species; its tandem duplicate, *ERVV-1*, is thought to be inactive due to disruptive mutations (Blaise *et al*. 2005, Esnault *et al*. 2013). *ERVPb-1* is another fusogenic *env*-derived gene that is conserved across various primate species, including humans (Blaise *et al*. 2005, McCarthy *et al*. 2020). Its protein is expressed in haematopoietic cell lineages, including a subset of macrophages (Matsuzawa *et al*. 2021). *ERVMER34-1* (*HEMO*) is expressed in the placenta and shed into the maternal blood circulation; although it has lost its fusogenic activity, it has been conserved under selection in simian primates, and the transmembrane protease BACE2 was recently identified as its interacting partner (Heidmann *et al*. 2017, Béguin *et al*. 2025).

The *env* genes are subject to dynamic turnover across different evolutionary lineages: some acquire functions, whilst others lose them. In previous studies, we termed this type of genetic shift the ‘baton pass hypothesis’, proposing that a large abundance of ERV-derived sequences in the genome provides multiple candidates with similar functional potentials, occasionally resulting in stochastic functional replacement (Fig.  1) (Imakawa *et al*. 2015). Recently, various primate genomes have been sequenced and deposited in the public database, providing whole-genome assemblies for 247 subspecies from 238 primate species, representing 81% of genera and 94% of families in the primate order (Kuderna *et al*. 2023, Kuderna *et al*. 2024). Leveraging this comprehensive genomic dataset enables investigating evolutionary changes in *env*-derived sequences with high resolution across species. In this study, utilizing 247 primate genome assemblies (Supplementary Table S1), we aimed to examine the evolutionary dynamics of *env*-derived open reading frames, including functional *env*-derived genes in humans. Based on the large-scale genomic survey, we identified evolutionary signatures characteristic of functional *env*-derived genes, and using this framework, we identified an *env*-derived gene candidate in tarsiers and experimentally confirmed its fusogenic activity.

## Materials and methods

### Ethics statement

The primate DNA samples used in this study were collected at the Japan Monkey Centre. This experiment is authorized by the ethics committee of Japan Monkey Centre under 2024009.

### PCR

To determine the nucleotide sequence of *ERVFRD-1* of *Papio hamadryas*, genomic DNA was first extracted from muscle tissue (Sample ID: Pr6767) and blood (Sample ID: 125) using the PureLink Genomic DNA Mini kit (K182001, Invitrogen). A PCR reaction was set up with the purified genomic DNA, employing the forward (5’-CCTCAAAGTAAGGTGATCCCAATA-3′) and reverse (5’-TATCCAGCCAGATCCACAGGAGA-3′) primers. Using KOD One polymerase (KMM-101, TOYOBO), the DNA was amplified for 30 cycles under the following conditions: denaturation at 98 °C for 10 s, annealing at 60 °C for 5 s, and extension at 68 °C for 20 s. The sequence of the amplicon was subsequently verified through Sanger sequencing (Eurofins Genomics).

### Genome assemblies

A total of 247 primate genome assemblies were used: 20 Hominoidea (apes), 87 Cercopithecoidea (catarrhine monkeys), 81 Platyrrhini (platyrrhine monkeys), 4 Tarsiiformes, and 55 Strepsirrhini. A complete list of the species and the accession numbers of their genome assemblies is provided in Supplementary Table S1. BEDtools v2.30.0 (Quinlan and Hall 2010) was used to extract genomic sequences.

### Detection of *env*-ORFs

ORFs that were at least 400 amino acids in length, beginning with a start codon and ending with a stop codon, were retrieved using the getorf programme from the European Molecular Biology Open Software Suite v6.5.7.0 (Rice *et al*. 2000). To remove ORFs that were generated by simple repeat sequences, ORFs with ˃30% of the sequence consisting of a single amino acid were excluded. ORFs with 10 or more consecutive ‘X’ amino acids were also excluded, to remove ORFs derived from ‘N’ sequences used to fill unknown bases or gaps in the genome assemblies. To identify *env*-ORFs from the retrieved ORFs, we employed the following three methods. First, we used the hmmscan programme from HMMER3 v3.2.2 (Eddy 2011) to search for Env domains with an expected threshold of 1E-10, using Env protein HMM profiles from the GyDB collection (Llorens *et al*. 2011). Second, a blastp search (E-value ≤1E-10) was performed using a set of representative human endogenous Env proteins, including ERVW-1/Syncytin-1 (GenBank ID: AAD14546.2), ERVFRD-1/Syncytin-2 (GenBank ID: NP_997465.1), ERVV-1 (GenBank ID: NP_689686.2), ERVV-2 (GenBank ID: NP_001177984.1), ERV3-1 (GenBank ID: NP_001007254.2), ERVPb-1 (GenBank ID: ABB52637.1), HEMO (GenBank ID: NP_001229619.1), HERV-T (GenBank ID: BAG06168.1), HERV-H (GenBank ID: AAD34324.1), and HERV-K115 (GenBank ID: AAL18259.1). Third, another blastp search (E-value ≤1E-10) was conducted using representative Env proteins of exogenous retroviruses (see Supplementary Table S2). The results from all three methods were then merged to create a non-redundant list of *env*-ORFs for each species.

### *env*-ORF clustering and phylogenetic analysis

The total set of *env*-ORFs from the primate genomes was clustered at a 40% amino acid sequence identity threshold using CD-HIT v4.8.1 (Li and Godzik 2006). Note that any singletons—ORFs that did not cluster with any other sequences—were excluded from this analysis. The amino acid sequences of representative *env*-ORFs in each cluster suggested by CD-HIT were aligned using MAFFT v7.487 with the ‘L-INS-i’ method (Katoh and Standley 2013). Following this, the resulting alignment was refined by removing gap-rich sites using the trimAl v1.4.rev15 programme with the ‘-gappyout’ option (Capella-Gutiérrez *et al*. 2009). The trimmed alignment was used for the phylogenetic tree construction using IQ-TREE2 v2.0.3 (Minh *et al*. 2020). To assess branch reliability, the ultrafast bootstrap values with 1000 replicates were computed for each node (Hoang *et al*. 2018).

### Plasmid construction

The ERVW-1/Syncytin-1 expression plasmid (phCMV3-Syn1 + 100) was previously constructed (Kitao *et al*. 2019). To construct the Env-Tar1 expression plasmid (phCMV3-Env-Tar1), dsDNA encoding the *env-Tar1* ORFs of *Carlito syrichta* was synthesized by Eurofins Genomics (Tokyo, Japan). The synthesized dsDNA was inserted into EcoRI and BamHI sites of phCMV3 by NEBuilder. For the Furin-cleavage inactive mutant, phCMV3-Env-Tar1 was linearized by PCR with the forward (5’-CGGTGGACAGCCTCAGTCTTTCACTGGTATG-3′) and reverse (5’-TGAGGCTGTCCACCGATCGACCAAATGAGGC-3′) primers and circularized by NEBuilder. For the FLAG-tagged mutant, the ORF with FLAG sequence was amplified from phCMV3-Env-Tar1 and phCMV3-Env-Tar1-R272A using the forward (5’-CGGTGGACAGCCTCAGTCTTTCACTGGTATG-3′) and reverse (5’-AACATCGTATGGGTAGGGTTTCGGGGACAACAG-3′) primers and inserted into phCMV3 using NEBuilder. All PCRs described above were carried out using KOD One Master Mix with a C1000 Touch thermal cycler (Bio-Rad).

### Western blotting

293T human embryonic kidney cells (RCB2202) were provided by the RIKEN BRC through the National BioResource Project of the MEXT, Japan. 293T cells were seeded on 100 mm dishes at 5 × 10^6^ cells per dish. The next day, cells were transfected with 10 μg of phCMV3, phCMV3-Syn2-FLAG (Shoji *et al*. 2023), phCMV3-Env-Tar1-FLAG, or phCMV3-Env-Tar1-R272A-FLAG, using 16 μL of Avalanche-Everyday Transfection Reagent (EZTEVDY-1, EZ Biosystems) per dish. Twenty-four hours after transfection, cells were fractionated using Minute Plasma Membrane/Protein Isolation and Cell Fractionation Kit (SM-005, Invent Biotechnologies). SDS/PAGE was performed, and peptides were transferred from the gel to polyvinylidene difluoride membranes. The membranes were incubated with an anti-FLAG antibody (F7425, Sigma-Aldrich), an anti-Na+/K + -ATPase antibody (ab7671, Abcam), and an anti-GAPDH antibody (MAB374, Sigma-Aldrich) as primary antibodies. The IRDye 800CW goat anti-mouse IgG secondary antibody (926-32210, LICORbio) and IRDye 680RD goat anti-rabbit IgG secondary antibody (926-68071, LICORbio) were used as a second antibody. Signals were detected by the Odyssey DLx Imaging System (LICORbio).

### Fusion assay

293T cells and HeLa-JVM cells (Moran *et al*. 1996, Nishimori *et al*. 2026) were plated on 24-well plates at 2 × 10^5^ cells/well and 1.5 × 10^5^ cells/well, respectively. On the following day, 0.5 μg of phCMV3, phCMV3-Syn1 + 100, phCMV3-Syn2 + 400 (Kitao *et al*. 2019), phCMV3-Env-Tar1, or phCMV3-Env-Tar1-R272A was transfected with 0.8 μl of Avalanche-Everyday Transfection Reagent. For the May-Grünwald-Giemsa stain, cells were stained with 300 μL of May-Grünwald solution (37126-14, Nacalai) and Giemsa solution diluted 10 times with water (37114-35, Nacalai) 24 h after transfection. For fusion-dependent LacZ assay, 293T cells as donors were seeded at 2 × 10^5^ cells per well on 24-well plates and were transfected with 0.5 μg of the env plasmid (phCMV3, phCMV3-Syn2 + 400, phCMV3-Env-Tar1 or phCMV3-Env-Tar1-R272A) and 0.5 μg of the LacZ reporter plasmid (pT7EMCV-LacZ), which expresses LacZ with internal ribosome entry site from encephalomyocarditis virus in the presence of T7 polymerase. The 293T cells as recipients were transfected with 0.5 μg of an expression plasmid of T7 polymerase (pCAG-T7Pol). Transfection was conducted with 0.8 μL of Avalanche Everyday Transfection Reagent per reaction. Donor and recipient cells were cocultured 6 h after transfection, and the LacZ-positive fused cells were stained by 5-bromo-4-chloro-3-indolyl-β-D-galactopyranoside (X-gal) 18 h after coculture.

### Similarity search for conserved *env*-ORFs

We conducted a tblastn (Camacho *et al*. 2009) search to find homologues of human ERV genes in primate genomes. We used tblastn 2.15.0 with parameters ‘-evalue 1e-5 -dbsize 3200000000 -outfmt 6 -num_alignments 500000 -show_gis -mt_mode 2 -num_threads 8 -seg no’. As queries, we used seven amino acid sequences of human ERV-related genes (Supplementary Table S3). We used 247 primate genome assemblies as a database (Supplementary Table S1).

## Results

### Variation of *env*-ORFs in primates

To identify nearly intact *env* gene-encoding ORFs from 247 primate genome assemblies (Supplementary Table S1), we first extracted all ORFs encoding 400 or more amino acids, defined from the initiation codon (i.e. ‘ATG’) to the stop codon. We then conducted protein domain searches using hidden Markov model (HMM) profiles obtained from the Gypsy database (Llorens *et al*. 2011). In parallel, we performed blastp searches using representative human ERV Env proteins and retroviral Env proteins (Supplementary Table S2). Then, the significant hits from both searches were combined, and long *env*-like ORFs, hereafter referred to as ‘*env*-ORFs’, were identified (Supplementary Fig. S1A).

**Figure 2:**
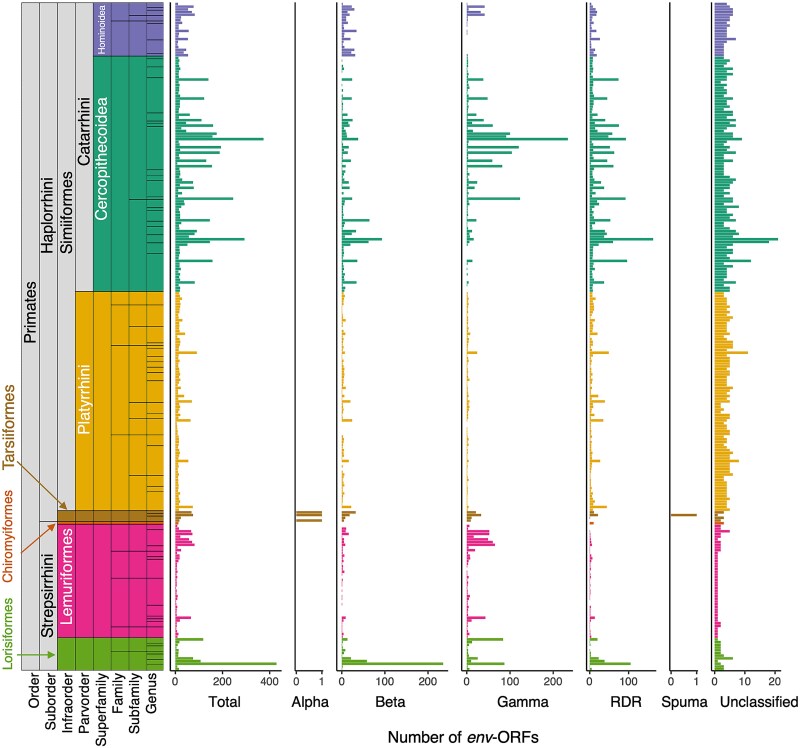
*env-ORFs in primate genomes.* The number of *env*-ORFs in the primate genome assemblies. The *env*-ORFs were classified into 5 groups based on the best hit to retroviral Env proteins. The source data for taxonomy and *env*-ORF counts are available in Supplementary Table S5.

All primate genome assemblies analyzed in this study contained at least three *env*-ORFs, resulting in a total of 8683 *env*-ORFs (Supplementary Fig. S1B**;**  Supplementary Table S4). Amongst the primates analyzed, *Nycticebus coucang* (Sunda slow loris) exhibited the highest number of *env*-ORFs, with 429 identified. We then classified these *env*-ORFs based on their best hit to known retroviral Env proteins (Fig.  2; Supplementary Table S5). The majority were assigned to the *Betaretrovirus* or *Gammaretrovirus* lineages. Notably, *env* genes belonging to the RD114-and-D-type-retrovirus (RDR) superfamily, which are found in both *Betaretrovirus* and *Gammaretrovirus* (Sinha and Johnson 2017), were counted separately. No *env*-ORFs related to *Deltaretrovirus* or *Epsilonretrovirus* were detected. Additionally, a number of unclassified *env*-ORFs showing no significant similarity to retroviral Env proteins by blastp were identified. *ERVMER34-1* (*HEMO*) was included in this unclassified group. An *env*-ORF related to *Spumaretrovirinae* (foamy viruses) was detected in *Cephalopachus bancanus* (Horsfield’s tarsier). Regarding this spumaretroviral *env*-ORF, *gag* and *pol* were also detected, and the 5′- and 3′-LTRs was almost identical (98.8% identity, Supplementary Fig. S2A). The phylogenetic tree of its reverse transcriptase also supports that this ERV belongs to *Spumaretrovirinae* (Supplementary Fig. S2B). ERVs of *Spumaretrovirinae* are rare in primate genomes, and an ERV with in-frame stop codons and frameshift mutations has been identified only in the aye-aye genome in primates (Han and Worobey 2012).

### Protein sequence clustering revealed different fates of *env*-ORFs

**Figure 3:**
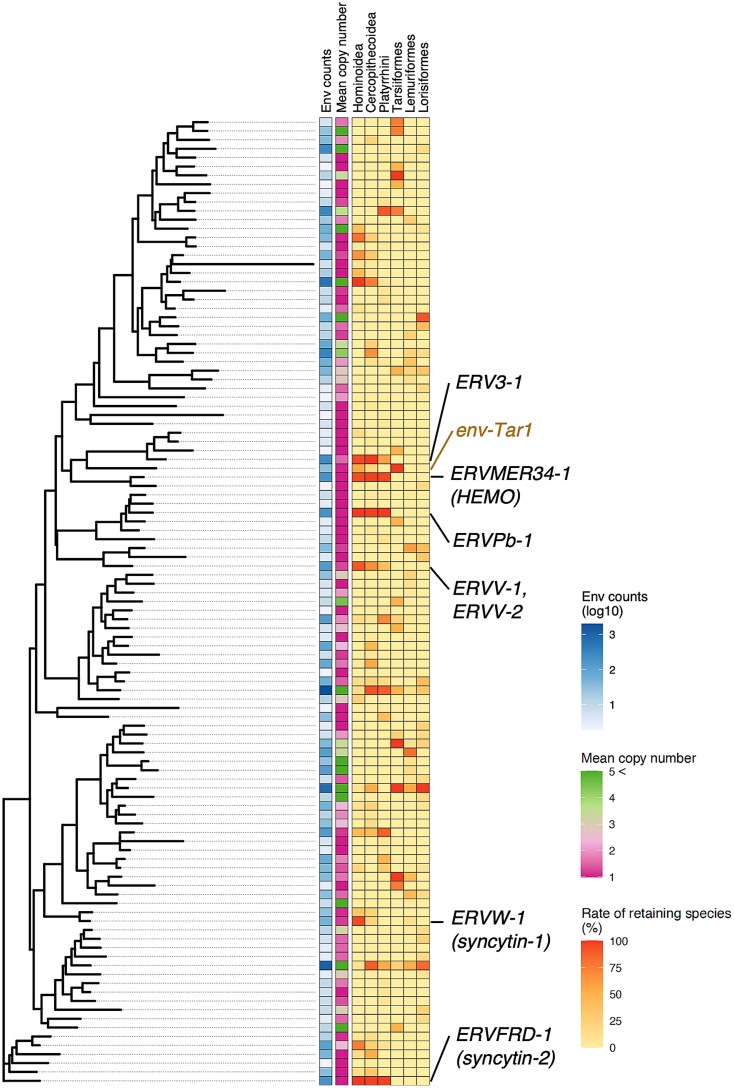
*Clustering of env-ORFs.* The 8683 *env*-ORFs were grouped into 109 clusters based on the amino acid sequence similarity. The left maximum-likelihood-based phylogenetic tree is constructed from the amino acid sequences of representative *env*-ORFs of clusters. ‘Env counts’ indicates the total number of *env*-ORFs in each cluster. ‘Mean copy number’ indicates the mean copy number of *env*-ORFs per genome assembly in each cluster. ‘Rate of retaining species’ indicates the proportion of species with at least one *env*-ORF in each taxonomic group. Clusters consisting of conserved single-copy *env* genes are expected to show a low ‘Mean copy number’ and a high ‘Rate of retaining species’. The representative conserved *env* genes are labelled on the clusters, including them. The tarsier ‘*env-Tar1*’ is described in Figs  4 and 5.

Next, we clustered the 8683 *env*-ORFs based on amino acid sequence similarity (40% sequence identity). The clustering yielded 109 clusters, and a phylogenetic tree was constructed using representative amino acid sequences from each cluster (Fig.  3). For each cluster, we calculated the proportion of primate species retaining *env*-ORFs in the cluster and the copy number per genome (Fig.  3 and Supplementary Fig. S3).

The phylogenetic tree shows that the known co-opted *env* genes (*ERV3-1*, *ERVMER34-1*, *ERVPb-1*, *ERVV-1*, *ERVV-2*, *ERVW-1*, and *ERVFRD-1*) were classified into separate clusters, excluding *ERVV-1* and *ERVV-2*. Each was included in a cluster present in many species with low copy numbers. For example, *ERVFRD-1*, which encodes Syncytin-2, was included in a cluster observed as a single copy in most species of Hominoidea, Cercopithecoidea, and Platyrrhini. Notably, *ERVV-1* and *ERVV-2* were observed in most of the species of Hominoidea and Cercopithecoidea but were poorly retained in Platyrrhini, although Mac-syncytin-3 encoded by *ERVV-2* exhibits fusogenic activity in several Platyrrhini species (Esnault *et al*. 2013). These functionally co-opted *env* genes were acquired in ancient evolutionary periods and have subsequently been conserved, which could explain their presence in many species with low copy numbers. Besides these known co-opted genes, we did not find any clusters with high retention rates and low copy numbers in Hominoidea, Cercopithecoidea, and Platyrrhini.

In Tarsiiformes (tarsiers), however, one *env*-ORF cluster showed high retention and a low copy number. No clusters exhibited signatures of co-option in Lemuriformes and Lorisiformes. These findings suggest that whilst most *env*-ORF groups exhibit copy number variation, those encoding co-opted proteins tend to undergo copy loss and eventually stabilize at a low copy number during evolution.

### Possible co-opted *env*-ORFs in Tarsiiformes

The clustering results suggested the presence of the *env*-ORF clusters specific to prosimian lineages (Fig.  3). To our knowledge, co-opted and long-term conserved *env* genes like *syncytin* genes remain to be elucidated in prosimians (i.e. Chiromyiformes, Lemuriformes, Lorisiformes, and Tarsiiformes). To search for the evolutionary conserved *env* gene in prosimians, we applied a more detailed phylogeny-based characterization for *env*-ORFs of prosimians. For Lemuriformes and Lorisiformes, one species from each genus was used for the phylogenetic analysis. In the phylogenetic tree of 217 *env*-ORFs from 13 species of Chiromyiformes (aye-aye) and Lemuriformes (lemurs), no shared single-copy orthologs were observed (Supplementary Fig. S4). Similarly, no single-copy ortholog was found from 604 *env*-ORFs in 7 species of Lorisiformes (galagos and lorises) (Supplementary Fig. S5). These data suggest that there is no strong evidence of conserved *env* genes inherited from the last common ancestor of Chiromyiformes, Lemuriformes, or Lorisiformes.

**Figure 4:**
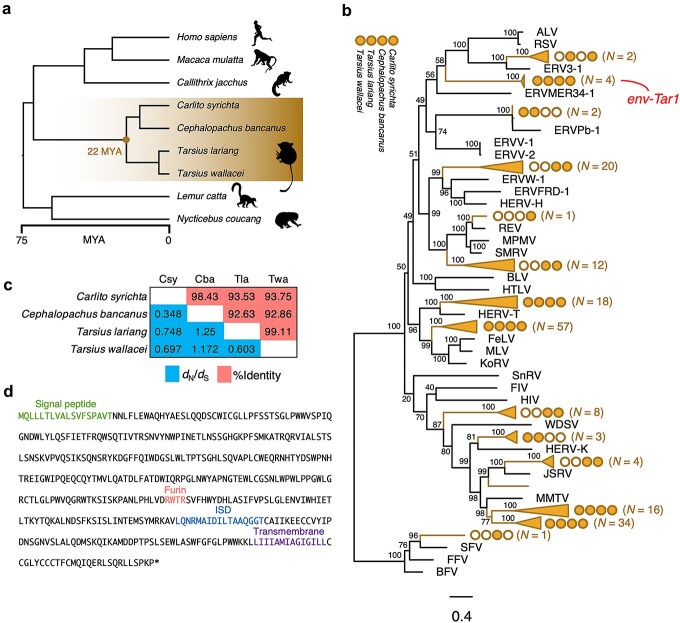
*Identification of a single orthologous env-ORF in Tarsiiformes.* (a) Species tree with estimated divergence time obtained from TimeTree (https://timetree.org/). (b) Maximum-likelihood-based phylogenetic tree reconstructed from representative retroviral Env proteins and tarsier *env*-ORF-encoded proteins comprising 183 sequences in total. The tips labelled with virus names represent the representative retroviruses used to infer phylogenetic relationship. Isosceles triangles indicate collapsed clades consisting exclusively of tarsier *env*-ORFs, and the depth (horizontal length) of each triangle reflects the branch lengths within the clade. The number of tarsiers’ *env*-ORFs (*N*) included in each collapsed clade is shown in parentheses. The four circles to the right side of each collapsed clade indicate whether each tarsier species has *env*-ORFs belonging to those collapsed clades: a filled circle indicates that the species has at least one *env*-ORF in the clade, whereas an open circle indicates that it does not. The order of the circles corresponds to the order of the species shown in the upper-left corner of the tree. The *env-Tar1* was the only *env*-ORF present in all four species, where it was retained as a single-copy ortholog. (c) Pairwise amino acid sequence identities and corresponding *d*_N_/*d*_S_ ratios amongst the *env-Tar1* ORFs. (d) Amino acid sequence of Env-Tar1 from *Carlito syrichta*. Characteristic retroviral Env features are highlighted. Each line shows 60 amino acids.

For Tarsiiformes, we analyzed genome assemblies of four species, which diverged ~22 million years ago (Fig.  4a). A phylogenetic tree of 183 *env*-ORFs of Tarsiiformes elucidated five *env*-ORF clades shared in all four tarsier species. Amongst them, four clades consisted of multi-copy *env*-ORFs, whereas one clade was retained as a single-copy ortholog (Fig.  4b). This gene was named *env-Tar1* (*env*-ORFs in Tarsiiformes 1). This *env-Tar1* was equivalent to the cluster shared in Tarsiiformes (Fig.  3). The *env-Tar1* ORFs encoded 446-448 amino acids, showing high pairwise identities (92.63%–99.11%) and a purifying selection (Fig.  4c). Furthermore, either intact *gag*-*pol* ORFs or LTRs were not detected in the surrounding genomic regions, suggesting the purifying selection to the *env-Tar1* gene (Supplementary Fig. S6). The putative Env-Tar1 protein possesses characteristic features of retroviral Env proteins, including an N-terminal signal peptide, a Furin cleavage site between the surface (SU) and transmembrane (TM) subunits, a hydrophobic transmembrane domain, and an immunosuppressive domain (Fig.  4d). These features suggest that the Env-Tar1 protein may retain cell fusion capability like the Syncytin proteins.

### Env-Tar1 is a fusogenic Env protein

**Figure 5:**
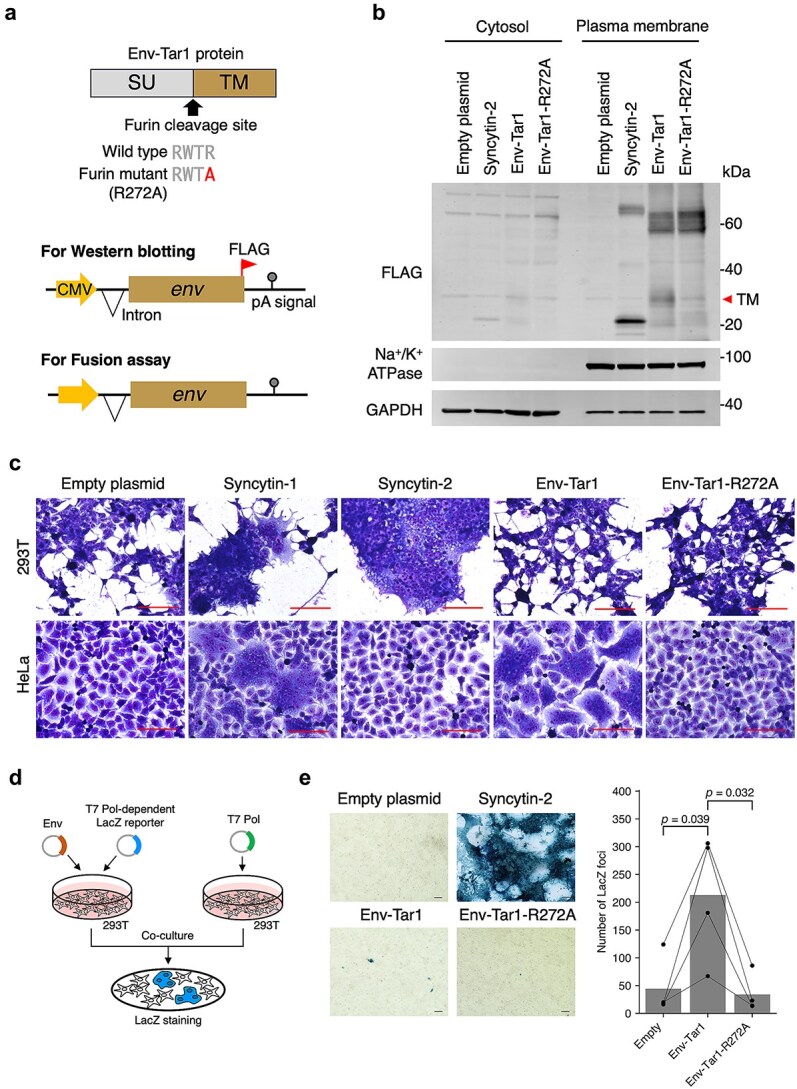
*The Env-Tar1 protein is a fusogen.* (a) Env-Tar1 possesses a Furin cleavage site that separates the surface (SU) and transmembrane (TM) subunits. A point mutation was introduced into the Furin cleavage site to generate a negative control (referred to as Env-Tar1-R272A). Env with a C-terminal FLAG tag was used for western blotting, whereas FLAG-free Env proteins were used for the cell fusion assay. (b) C-terminal FLAG-tagged Env proteins were expressed in 293T cells, and cytosol and plasma membrane fractions were subjected to western blotting. FLAG-tagged Syncytin-2, Env-Tar1, and Env-Tar1-R272A were localized in the plasma membrane. The TM subunit was absent in the Env-Tar1-R272A. (c) Env-transfected cells were visualized by May-Grünwald-Giemsa stain. The scale bars represent 100 μm. (d) Cell-fusion-dependent LacZ assay. In donor 293T cells, the Env expression plasmid was co-transfected with a reporter plasmid that expresses lacZ in the presence of T7 polymerase. Recipient 293T cells were transfected with a plasmid expressing T7 polymerase. After co-culture of donor and recipient cells, fused cells expressing LacZ were visualized by X-gal staining. (e) LacZ signals by cell fusion. The wild-type Env-Tar1 induced more LacZ-positive foci than the empty vector or the Furin mutant. The scale bars represent 100 μm. In the right panel, bars represent mean values, and each dot indicates an individual experiment. Data points connected by lines denote samples analyzed within the same experimental replicate the *P*-value was calculated by the paired t-test using scipy.stats.ttest_rel (SciPy, version 1.15.2).

We experimentally evaluated whether the Env-Tar1 protein functions as a fusogen. We constructed the plasmid for expressing the Env-Tar1 protein of *Carlito syrichta* (Philippine tarsier). As a negative control, we generated a Furin cleavage-inactive mutant in which the arginine at position 272 was replaced with alanine (Env-Tar1-R272A) (Fig.  5a). For protein detection by western blotting, the C-terminal FLAG tag was fused to the Env protein, whereas FLAG-free Env proteins were used for the cell fusion assay (Fig.  5a). The Env-Tar1-FLAG protein localized to the plasma membrane in the same manner as the Syncytin-2-FLAG protein in 293T cells (Fig.  5b). The signal of TM subunit was detected in the Env-Tar1-FLAG, but not in the Env-Tar1-R272A-FLAG (Fig.  5b). We next examined the cell fusion ability by transfecting plasmids encoding FLAG-free Env protein into 293T and HeLa cells. Syncytin-1 exhibited significant cell fusion in both 293T and HeLa cells, whilst Syncytin-2 induced cell fusion only in 293T cells (Fig.  5c). This is consistent with previous report (Blaise *et al*. 2003). Env-Tar1 was highly fusogenic in HeLa cells but not in 293T cells (Fig.  5c). Env-Tar1-R272A did not cause cell fusion in either cell line (Fig.  5c). To verify the possibility of weak cell fusion by Env-Tar1 in 293T cells, we performed a sensitive fusion-dependent LacZ assay (Kitao *et al*. 2023) (Fig.  5d). Although the LacZ signal induced by Env-Tar1 was much weaker than by Syncytin-2, a significant increase in LacZ foci was observed with wild-type Env-Tar1 compared to the empty plasmid and Env-Tar1-R272A (Fig.  5e). These data demonstrate that Env-Tar1 functions as a fusogen and the cleavage of Furin site is important for its membrane fusion ability.

### Possible truncation of conserved *env*-ORFs in various species

**Figure 6:**
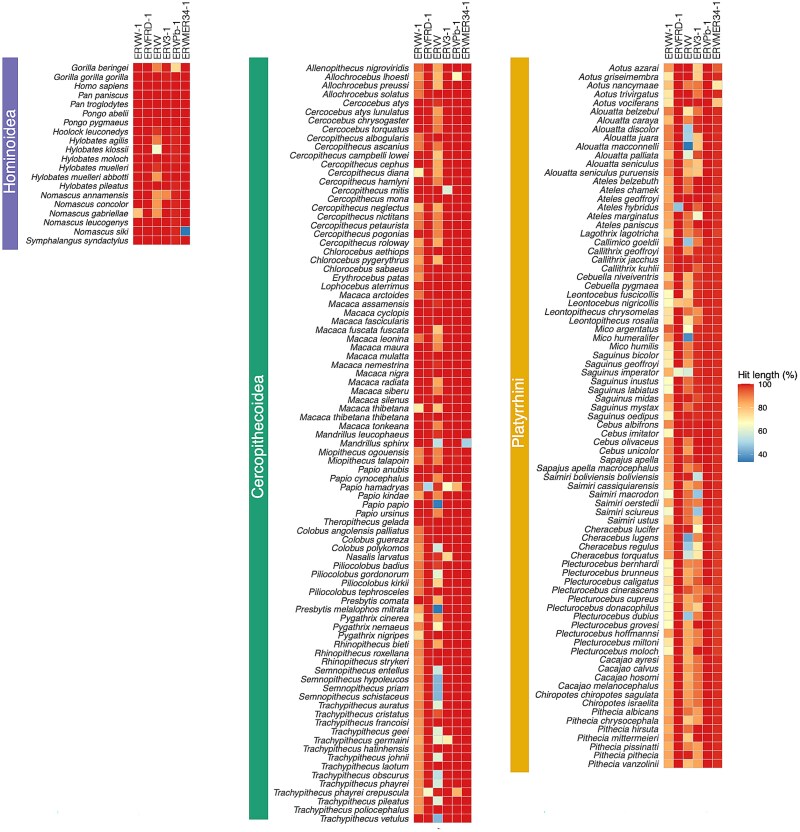
*Conservation of the human env gene in primate genomes.* We performed tblastn against primate genome assemblies using the human *env* genes, which are known to be evolutionarily conserved, as queries. We then examined the coverage of each query *env* gene by the best hits. Query coverage in each genome assembly is shown as a heat map.

As described above, clusters of co-opted *env* genes are often widely shared amongst species. However, upon closer inspection of the clustering results, we found that some species lacked *env*-ORFs that are expected to be conserved. This suggests that certain co-opted *env* genes may have lost their function within specific lineages. To investigate this possibility, we examined conservation of seven known human *env* genes: *ERVW-1* (*syncytin-1*), *ERVFRD-1* (*syncytin-2*), *ERVV-1*, *ERVV-2*, *ERV3-1*, *ERVPb-1*, and *ERVMER34-1* (*HEMO*). We performed the tblastn search using these seven genes as queries and investigated their ORF length (Supplementary Table S3). The results suggest that several *env* gene ORFs are truncated in multiple species across various lineages (Fig.  6**;**  Supplementary Table S6).

In particular, we found various truncations in *ERVV* ORFs. Since *ERVV-1* and *ERVV-2* are tandemly duplicated genes and their sequences are highly similar to each other (Kjeldbjerg *et al*. 2008, Esnault *et al*. 2013), we obtained sequences that showed the highest similarity to either *ERVV-1* or *ERVV-2*. Nevertheless, several species were found to possess only short ORFs (Fig.  6). In *Macaca mulatta* (rhesus macaque), *ERVV-2* is fusogenic and also referred to as *Mac-syncytin-3* (Esnault *et al*. 2013); however, it was found that the ORFs are shortened in multiple Cercopithecoidea species. Amongst them, the contigs of three *Semnopithecus* species (*Semnopithecus hypoleucos*, *Semnopithecus priam*, and *Semnopithecus schistaceus*) were truncated at the same position, resulting in no intact *ERVV* ORFs being detected (Supplementary Fig. S7), which may suggest that deletion events in the *ERVV* family have occurred in the ancestor of the lineage. Although we cannot rule out the possibility that the apparent truncations are due to simply low genome assembly accuracy (see Discussion), these results suggest that species-specific truncations may have occasionally occurred for *env*-derived genes during primate evolution.

## Discussion

In this study, we found that primate *env*-ORFs are mainly derived from *Betaretrovirus* and *Gammaretrovirus*, and that the number of *env*-ORFs in primates varies greatly, even within the same genus. These results suggested that the turnover of *env*-ORFs in primate genomes is highly active, illustrating the dynamic evolution of *env* genes in primates. On the other hand, we found that *env*-derived genes that have acquired functions are conserved across many lineages, and related sequences are maintained at low copy numbers in each species. Indeed, using this evolutionary framework, we identified an *env*-derived gene candidate in Tarsiiformes, named *env-Tar1*, that retained membrane fusion activity. Since *env-Tar1* is shared amongst all four tarsiers and was not detected in simians, the insertion most likely occurred after the divergence of Tarsiiformes from the other haplorhines, placing it within a broad window of roughly 22–69 MYA. Although it is difficult to estimate the exact insertion time, this timeframe is sufficiently long for the *gag* and *pol* genes to lose their ORFs because they are presumed to lack functional constraint. Blanco-Melo *et al.* simulated that, under neutral evolution, only 6% of *env* sequences retained an intact ORF over 13-19 million years (Blanco-Melo *et al*. 2017). Indeed, in each of the previously reported co-opted human *env*-derived genes, only the *env* ORF has been preserved, whereas the flanking *gag* and *pol* genes have been degraded by stop codons, frameshifts, or deletions: *ERVW-1/syncytin-1* (Bonnaud *et al*. 2005), *ERVFRD-1/syncytin-2* (Blaise *et al*. 2003), *ERVV-1/ERVV-2* (Kjeldbjerg *et al*. 2008), *ERV3-1* (de Parseval and Heidmann 1998), *ERVPb-1* (Blaise *et al*. 2005), and *ERVMER34-1/HEMO* (Heidmann *et al*. 2017). However, we also found that some *env* genes that had once acquired functions may have subsequently lost them, suggesting that these *env* genes may have been functionally replaced.

Indeed, in ruminants, *syncytin-Rum1* was reported to encode a fusogenic protein and be highly expressed in the placenta (Cornelis *et al*. 2013). However, in the Bovidae family, *fematrin-1*, another *env*-derived gene found only in Bovidae, shows higher expression levels and greater fusion capacity than *syncytin-Rum1* (Nakaya *et al*. 2013). This likely indicates that *fematrin-1* has replaced the molecular function related to the cell–cell fusion process of *syncytin-Rum1* in Bovidae (Imakawa *et al*. 2015, Nakaya and Miyazawa 2015, Imakawa *et al*. 2022). Our findings also support the idea that functional *env*-derived gene replacements may frequently occur during mammalian evolution.

Why do *env*-derived genes change so dynamically during evolution? One possible explanation is the shared usage of receptors of host cells by viruses and ERVs. For example, it is known that envelope proteins of RDR interference group viruses, such as Syncytin-1, Env-Tac1, suppressyn, and RD-114, use host ASCT-2 protein on the surface of cells as their receptor (Marin *et al*. 2000, Sugimoto *et al*. 2013, Kitao *et al*. 2023, Štafl *et al*. 2024). For *ASCT-2* gene, it has been reported that independent insertion mutations occurred in rodents and birds at the sites where these proteins interact with viral envelope proteins (Miyaho *et al*. 2015). Indeed, many positively selected sites have been detected in receptor genes of primates, particularly at the virus-receptor interfaces (Wang *et al*. 2020). This suggests that even if *env*-derived genes can functionally utilize membrane fusion activity, mutations in the host receptors may be advantageous to the host if infectious viruses use the same receptor. This may contribute to the continuous turnover of *env*-derived genes during evolution. In fact, various *env*-derived genes are known to be under purifying selection (Cornelis *et al*. 2013, Shoji *et al*. 2023), which is consistent with our finding that they tend to be maintained at low copy numbers. Nevertheless, the fact that they still undergo frequent changes may be due to positive selection acting on their receptors, which are involved in viral escape. In contrast, retroviral proteins derived from genes other than *env,* such as *PEG10* and *PEG11*/*RTL1,* have been reported to be functionally conserved across many mammalian species (Suzuki *et al*. 2007, Shiura *et al*. 2023). Therefore, host-virus arms races may also drive dynamic changes in *env*-derived genes during evolution.

Note that potential genome misassemblies may have led to misannotations of truncations in some *env*-derived genes. Indeed, we found that genomes with shorter contigs tend to contain fewer *env*-derived ORFs (Supplementary Fig. S8). Further, *ERVW-1*, which encodes Syncytin-1 and is expected to be conserved in Hominoidea, appears truncated in *Nomascus gabriellae* (yellow-cheeked gibbon) (Fig.  6). Two syncytin-1-like ORFs found in this species contain nonsense mutations (Supplementary Fig. S9A), suggesting that they may have lost *ERVW-1* although we cannot rule out the possibility that this is an artefact resulting from the low quality of the genome assembly. For *ERVFRD-1* encoding Syncytin-2, most simians retain intact ORFs, but six species (two Cercopithecoidea and four Platyrrhini species) carry truncated forms that seem to have occurred independently. In *P. hamadryas* (Hamadryas baboon), the reference genome shows a 1-bp deletion causing ORF truncation, yet PCR analyses from two individuals revealed no such deletion (Supplementary Fig. S9B). Comparative analyses further indicate that the reference sequence likely represents a chimeric artefact involving another ERV copy on chromosome 3 (Supplementary Fig. S9C). These findings suggest that several apparent truncations of *syncytin* genes may result from genome misassemblies.

On the other hand, a shortened *env*-derived gene does not necessarily mean that the gene has lost its function. In fact, several truncated *env*-derived genes have been reported to retain biological activity, such as *suppressyn* and others in primates (Sugimoto *et al*. 2013, Frank *et al*. 2022, Miyake *et al*. 2022) and *Refrex-1* in cats (Ito *et al*. 2013). However, their functions differ from the original membrane fusion ability of Env proteins, as they act by interfering with host receptors to block viral infection. Since potential functions such as interference-mediated inhibition of viral infection were not examined in this study, the precise roles of these truncated *env*-derived genes remain largely unclear. In addition, since the Env queries used in this study were limited to HERVs and reference retroviruses (Supplementary Table S2), it remains possible that additional, as yet unidentified, *env*-ORFs exist in primate genomes.

Although there are several limitations, our study indicates that *env*-derived genes exhibit considerable diversity, and functional genes may also undergo frequent changes in primates. As the accuracy of genome sequences improves and more genomes of various species and individuals become accessible, our understanding of this area will advance even further.

## Supplementary Material

Supplementary_materials_veag044

Virus-Evolution_Supporting_information_veag044

## Data Availability

The raw datasets, including supplementary tables, generated in this study are available on Zenodo: https://zenodo.org/records/17706639.
