# Supplementary material for: Dynamic evolution of retroviral envelope-derived sequences in primates
Source: Virus Evol. 2026 Jul 16;12(1):veag044. doi: 10.1093/ve/veag044 (PMC13461226; doi:10.1093/ve/veag044)
Supplement: Supplementary file 1 [file virus-evolution_supporting_information_veag044.docx]

Fig. S1. Identification of *env*-ORFs in 247 primate genome assemblies. (A) Workflow for identifying *env*-ORFs in primate genome assemblies. We obtained *env*-ORFs by three methods of similarity search for retroviral reference databases. (B) Venn diagram illustrating the *env*-ORFs identified by three methods. In total, 8,683 *env*-ORFs were obtained.

Fig. S2. The endogenized spumaretrovirus harboring the *env*-ORFs in *Cephalopachus bancanus*. (A) ORFs of the proviral sequence. The half-length vertical bars indicate start codons, and full-length vertical bars indicate stop codons. ORFs of at least 300 nt were filled. ORFs from a stop codon to a stop codon are shown at the top (pink), and ORFs from an ATG triplet to a stop codon are shown at the bottom (light blue). ORFs of *gag*, *pol*, and *env* were indicated. 5′- and 3′-LTRs were filled with gray. (B) Phylogenetic tree of reverse transcriptase domains. The endogenized spumaretrovirus in *Cephalopachus bancanus* is indicated by an arrow*.*

Fig. S3. Clustering of *env*-ORFs. The x-axis represents the sequence identity established via clustering. The y-axis represents the number of clusters and also shows the original number of sequences before clustering. The “Multi_cluster” is comprised of more than two sequences and the “Solo_cluster” consists of one sequence. A logarithmic scale was used on the y-axis. The phylogenetic tree of Figure 3 was constructed by representative sequences of clusters with 40% identity.

Fig. S4. Maximum-likelihood-based phylogenetic tree reconstructed from representative retroviral Env proteins and 217 Env proteins encoded in env-ORFs of Chiromyiformes (1 species) and Lemuriformes (12 species). (A) Species tree with estimated divergence time obtained from TimeTree (<https://timetree.org/>). (B) The virus names labeling the tips indicate the representative retroviruses used to infer phylogenetic relationship. Isosceles triangles indicate collapsed clades consisting exclusively of *env*-ORFs, and the depth (horizontal length) of each triangle reflects the branch lengths within the clade. The number of *env*-ORFs (*N*) included in each collapsed clade is shown in parentheses. The 13 circles to the right side of each collapsed clade indicate whether each species has *env*-ORFs belonging to those collapsed clades: a filled circle indicates that the species has at least one *env*-ORF in the clade, whereas an open circle indicates that it does not. The order of the circles corresponds to the order of the species shown in the lower-left corner of the tree.

Fig. S5. Maximum-likelihood-based phylogenetic tree reconstructed from representative retroviral Env proteins and 607 Env proteins encoded in *env*-ORFs of Lorisiformes (7 species). (A) Species tree with estimated divergence time obtained from TimeTree (<https://timetree.org/>). (B) The tips labeled with virus names represent the representative retroviruses used to infer phylogenetic relationship. Isosceles triangles indicate collapsed clades consisting exclusively of *env*-ORFs, and the depth (horizontal length) of each triangle reflects the branch lengths within the clade. The number of *env*-ORFs (*N*) included in each collapsed clade is shown in parentheses. The seven circles to the right side of each collapsed clade indicate whether each species has *env*-ORFs belonging to those collapsed clades: a filled circle indicates that the species has at least one *env*-ORF in the clade, whereas an open circle indicates that it does not. The order of the circles corresponds to the order of the species shown in the lower-left corner of the tree.

Fig. S6. Distribution of ORFs within 5 kbp upstream and 2 kbp downstream of the *env-Tar1* ORF in *Carlito syrichta*. Full length vertical lines indicate stop codons in each reading frame, while half-length vertical lines indicate start codons. ORFs longer than 180 codons are highlighted. The upper section (pink) shows ORFs spanning from stop codon to stop codon, while the lower section (blue) shows ORFs spanning from start codon to stop codon.

**Fig. S7. Multiple alignment of nucleotide sequences of ERVV from humans and three *Semnopithecus* species.** For the Semnopithecus species, each contig was obtained with the following accession numbers: *S. hypoleucos*, CAUYNQ010114950.1; *S. priam*, CAUYPN010033499.1; *S. schistaceus*, CAUYPV010179808.1.

**Fig. S8. A scatter plot illustrating the relationship between N50 length of contigs and the count of env ORFs (≥400 amino acids) across primate species.**

**Fig. S9. Variation of *ERVW-1* (*syncytin-1*) and *ERVFRD-1* (*syncytin-2*) with truncating mutations in genome assemblies.** (A) The intact ORF encoding Syncytin-1 was not identified in the *Nomascus gabriellae* genome assembly (GCA_963574555.1). *ERVW-1*-like sequences were still identified; however, these two ORFs were shorter than the intact *syncytin-1* ORF and were located in short contigs. (B) The *ERVFRD-1* encoding Syncytin-2 was truncated by mutations in the *Papio hamadryas* genome assembly (GCA_023781915.1). We performed PCR for *ERVFRD-1* from the *P. hamadryas* genomic DNA. The PCR products were electrophoresed on a 1% agarose and were subjected to Sanger sequencing to determine its nucleotide sequence. The amplified sequences encoded a complete ORFs for Syncytin-2. (C) Analysis of the genomic region corresponding to *ERVFRD-1* in the *P. hamadryas* genome assembly. A single-base deletion was identified within the *ERVFRD-1* ORF, resulting in the loss of the open reading frame. The sequences flanking this deletion were compared with those of the closely related species *P. anubis*.

Supplementary Table legends

**Supplementary Table S1.** List of 247 primate genome assemblies used in this study.

**Supplementary Table S2**. Queries used for the *env*-ORF search.

**Supplementary Table S3.** List of seven human Env gene IDs used for the tblastn search.

**Supplementary Table S4.** Annotations for *env*-ORFs. Species name, genome assemblies, location on the assemblies, and the hit queries.

**Supplementary Table S5.** The number of *env*-ORFs for each genome assembly.

**Supplementary Table S6.** Coverage of the best hits for human *env*-derived genes in the genome assemblies.
